# Flagellar membranes are rich in raft-forming phospholipids

**DOI:** 10.1242/bio.011957

**Published:** 2015-08-14

**Authors:** Mauro Serricchio, Adrien W. Schmid, Michael E. Steinmann, Erwin Sigel, Monika Rauch, Daria Julkowska, Serge Bonnefoy, Cécile Fort, Philippe Bastin, Peter Bütikofer

**Affiliations:** 1 Institute of Biochemistry & Molecular Medicine, University of Bern, Bern 3012, Switzerland; 2 Proteomics Core Facility, École Polytechnique Fédérale de Lausanne, Lausanne 1015, Switzerland; 3 Trypanosome Cell Biology Unit, Pasteur Institute and INSERM U1201, Paris 75015, France

**Keywords:** Flagella, Lipid rafts, Membrane lipids, Mass spectrometry, Sphingolipids, *Trypanosoma brucei*

## Abstract

The observation that the membranes of flagella are enriched in sterols and sphingolipids has led to the hypothesis that flagella might be enriched in raft-forming lipids. However, a detailed lipidomic analysis of flagellar membranes is not available. Novel protocols to detach and isolate intact flagella from *Trypanosoma brucei* procyclic forms in combination with reverse-phase liquid chromatography high-resolution tandem mass spectrometry allowed us to determine the phospholipid composition of flagellar membranes relative to whole cells. Our analyses revealed that phosphatidylethanolamine, phosphatidylserine, ceramide and the sphingolipids inositol phosphorylceramide and sphingomyelin are enriched in flagella relative to whole cells. In contrast, phosphatidylcholine and phosphatidylinositol are strongly depleted in flagella. Within individual glycerophospholipid classes, we observed a preference for ether-type over diacyl-type molecular species in membranes of flagella. Our study provides direct evidence for a preferential presence of raft-forming phospholipids in flagellar membranes of *T. brucei*.

## INTRODUCTION

Eukaryotic flagella allow movement of single-celled organisms. Most flagella are composed of a central cylindrical structure, the axoneme, made of nine doublets of microtubules surrounding a central microtubule pair. The axoneme is attached to the basal body and is surrounded by a membrane. Proteomic studies of flagella isolated from various organisms have revealed a remarkable complexity, with more than 500 different proteins identified in certain species, yet many proteins involved in flagellum architecture and motility are highly conserved between eukaryotes (reviewed in Vincensini et al., 2011). In contrast, little is known about the composition and properties of the flagellar membrane.

Many important discoveries in the field of flagellum research have been made in unicellular organisms, such as *Chlamydomonas*, *Paramecium*, *Tetrahymena* and *Trypanosoma* (reviewed in Vincensini et al., 2011). Trypanosomes are protozoan parasites causing several tropical diseases, including human African sleeping sickness, a fatal disease caused by *T. brucei* parasites threatening the health of millions of people in rural sub-Saharan Africa (Kennedy, 2013). However, *T. brucei* is not only an important pathogen but has also emerged as model organism to study various biological processes. In particular, due to the availability of different parasite species and different life cycle stages in cultures growing to high densities, completely sequenced genomes, and a wide selection of tools for molecular biology and reverse genetics, *T. brucei* has been chosen as model organism to study flagellum biology. In *T. brucei*, the flagellum emerges from the cell body through the flagellar pocket, a depression of the cell membrane, and is anchored to the cell body for most of its length. It plays an important role in parasite morphogenesis and pathogenicity (reviewed in Langousis and Hill, 2014; Vaughan, 2010). The flagellar membrane is in continuity with that of the flagellar pocket and, because of its attachment alongside the parasite, is also in very close proximity to the membrane of the cell body. Despite that, the protein and lipid compositions of these membranes do not appear to be identical (Hanrahan et al., 2009; Oberholzer et al., 2011; Tetley, 1986).

Trypanosomes have also been used to study eukaryotic lipid synthesis and lipid biology (Serricchio and Bütikofer, 2011). The *T. brucei* lipid composition and the underlying biosynthetic pathways have been investigated in great detail (reviewed in Ramakrishnan et al., 2013; Smith and Bütikofer, 2010). Some of the most striking characteristics of the lipid composition of *T. brucei* include (a) the presence of high amounts of ether-type phospholipid molecular species, especially in the glycerophospholipid classes phosphatidylethanolamine (PE) and phosphatidylserine (PS) (Patnaik et al., 1993; Richmond et al., 2010), (b) a life cycle-dependent synthesis of sphingophospholipid classes, resulting in the presence of inositol phosphorylceramide (IPC) in procyclic (the proliferative stage found in the midgut of the insect vector, the tsetse fly) and ethanolamine phosphorylceramide (EPC) in proliferating bloodstream form parasites (Sutterwala et al., 2008), and (c) the ability of trypanosomes to take up and incorporate sterols from the host environment (Dixon et al., 1971, 1972).

It has been reported that flagellar membranes are enriched in raft-type lipids, i.e. sterols in quail oviduct (Chailley and Boisvieux-Ulrich, 1985) and sphingophospholipids in *Paramecium* (Kaneshiro et al., 1984). In addition, freeze-fracture electron microscopy studies in *Leishmania* and *Trypanosoma* parasites showed a higher density of filipin-induced lesions on the flagellar membrane compared to the adjacent cell body membrane, indicative of a higher 3-β-hydroxysterol content in the flagellum (De Souza, 1995; Tetley, 1986; Tetley et al., 1986). Further evidence supporting the presence of raft-type lipids in flagella has been provided by Laurdan microscopy studies in *T. brucei*, showing that lipids in the flagellar membrane are in a more ordered state than in the cell body membrane (Tyler et al., 2009). In addition, using immunofluorescence microscopy, glycosphingolipids were shown to preferentially localize to flagella in *T. brucei* procyclic forms (Tyler et al., 2009). Finally, a member of the calflagin protein family has been shown to localize preferentially in the *T. brucei* flagellum by associating with raft-typical detergent-resistant domains (Maric et al., 2011). However, to our knowledge a detailed analysis of the flagellar membrane lipid composition of *T. brucei*, or any other eukaryote, is not available.

**Abbreviations**

Cer: ceramide
CID: collision-induced dissociation
CL: cardiolipin
LC-MS/MS: liquid chromatography tandem mass spectrometry
IPC: inositol phosphorylceramide
PC: phosphatidylcholine
PE: phosphatidylethanolamine
PG: phosphatidylglycerol
PI: phosphatidylinositol
PS: phosphatidylserine
RT: retention time
SM: sphingomyelin

Recently, intact flagella have been separated mechanically from trypanosome mutants that have defects in flagellum adhesion (Oberholzer et al., 2011; Subota et al., 2014). In the present study, we have used a similar approach to obtain pure intact flagella and applied a novel liquid chromatography high-resolution mass spectrometry method to determine their phospholipid composition compared to that of whole cells. Our results show that two glycerophospholipid classes, phosphatidylcholine (PC) and phosphatidylinositol (PI), are depleted in flagella, whereas PE, PS, ceramide and the sphingophospholipid classes IPC and sphingomyelin (SM) are enriched in flagella compared to whole cells. In addition, we found a strong enrichment of ether-type molecular species of PE, PC and PS in flagella. Our results demonstrate for the first time that the *T. brucei* flagellum is enriched in raft-type lipids, in particular ether-type glycerophospholipid species, suggesting the existence of a distinct membrane environment.

## RESULTS AND DISCUSSION

### Flagella isolation after knockdown of Tb927.10.2880 expression

**Fig. 1.**
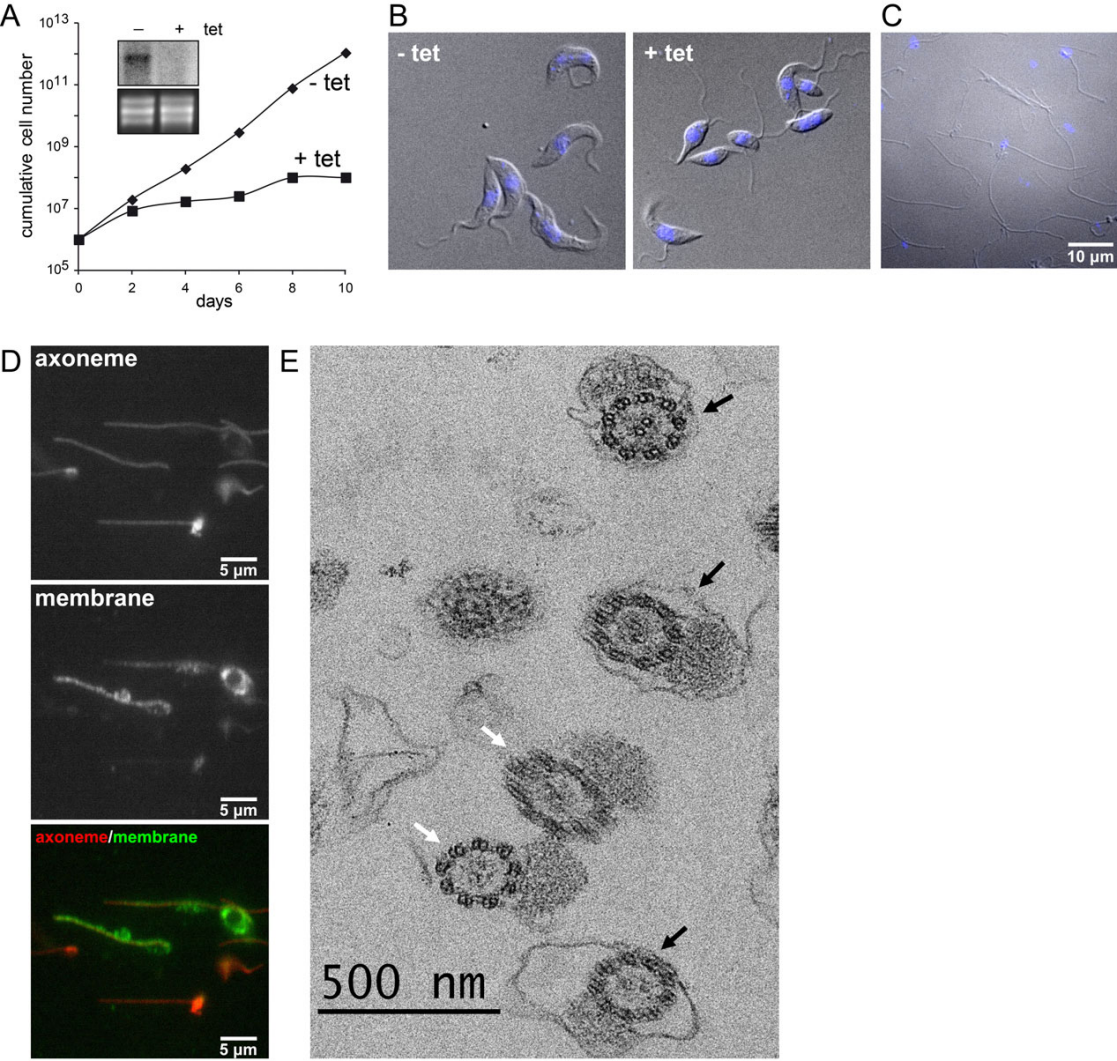
**Isolation of pure flagella.** (A) Growth curve of trypanosomes upon tetracycline-induction of double-stranded RNA targeting Tb927.10.2880. Knockdown of Tb927.10.2880 results in a growth defect after 2 days of induction. The inset shows the Northern blot analysis confirming the disappearance of the corresponding mRNA upon induction (top) and the ethidium bromide stained rRNA as loading control (bottom). (B) Differential interference contrast (DIC) micrographs of uninduced (− tet) and RNAi induced (+ tet) parasites. After 1 day of induction, flagella appear detached from the cell bodies. (C) DIC micrograph of isolated flagella. Mitochondrial and nuclear DNA was stained with DAPI, imaged by fluorescence microscopy and is shown in blue. Scale bar: 10 µm. (D) Flagella were spread on slides, fixed and processed for double immunofluorescence with the axoneme marker Mab25 (top panel) and with anti-calflagin as a membrane marker (middle panel). Merged images are shown at the bottom as indicated. Flagella commonly tend to curve under these conditions. Scale bars: 5 µm. (E) Flagellar preparations were fixed, sectioned and examined by transmission electron microscopy. Recognisable elements were overwhelmingly flagella and half of them possessed a membrane (black arrows). Axonemes apparently deprived of their membrane are indicated with a white arrow. Scale bar: 500 nm.

In *T. brucei*, the flagellum extends along and is closely attached to the cell body (Kohl and Bastin, 2005). It has previously been shown that RNAi-mediated down-regulation of expression of a putative calcium channel protein (Tb927.10.2880) in *T. brucei* bloodstream forms resulted in detachment of the flagellum from the cell body (Oberholzer et al., 2011). We now show that a similar phenotype is also observed in procyclic forms. Tetracycline-inducible expression of a hairpin RNA targeting Tb927.10.2880 mRNA led to its strong depletion as confirmed by Northern blotting (Fig. 1A, inset), resulting in a strong growth defect after two days of culture (Fig. 1A), a feature noted for most mutants where flagellum adhesion is compromised (LaCount et al., 2002; Rotureau et al., 2014; Sun et al., 2013; Sunter et al., 2015; Zhou et al., 2010, 2015). Down-regulation of Tb927.10.2880 expression resulted in detachment of the flagellum (Fig. 1B), allowing its release from the parasite body by use of mechanical forces and subsequent isolation of detached flagella using previously published procedures (Oberholzer et al., 2011; Subota et al., 2014). Examination by light microscopy demonstrated that the preparation contains pure flagella, with no visible contamination by intact trypanosomes or remnant cell bodies (Fig. 1C). Approximately 30–50% of purified flagella contained kinetoplasts (mitochondrial DNA) attached to the basal bodies of flagella. In trypanosomes, the mitochondrial DNA is physically linked to the basal body by specific cytoskeletal structures (Ogbadoyi et al., 2003; Robinson and Gull, 1991). To evaluate the quality of the preparation, samples were double stained with antibody markers for the axoneme (MAP6-related protein) (Dacheux et al., 2012) and for the flagellar membrane (calflagins) (Engman et al., 1989). All flagella appear positive for the anti-axoneme marker whereas some but not all were strongly positive for the membrane marker (Fig. 1D). The rest of the population produced weaker, yet positive signals. Next, the preparations were fixed and analysed by transmission electron microscopy (Fig. 1E). The vast majority of the sections were across flagella and very few contaminants were observed, confirming the high enrichment in flagella. Closer examination revealed that 54% of the axonemes (black arrows) were wrapped by a membrane (*n*=81). We regularly noticed the presence of membrane “remnants” that may have come off the flagella during the purification process. Overall, these results demonstrate the quality of the flagella preparations, with more than half of the flagella possessing an intact membrane.

### High-resolution liquid chromatography mass spectrometry

**Fig. 2.**
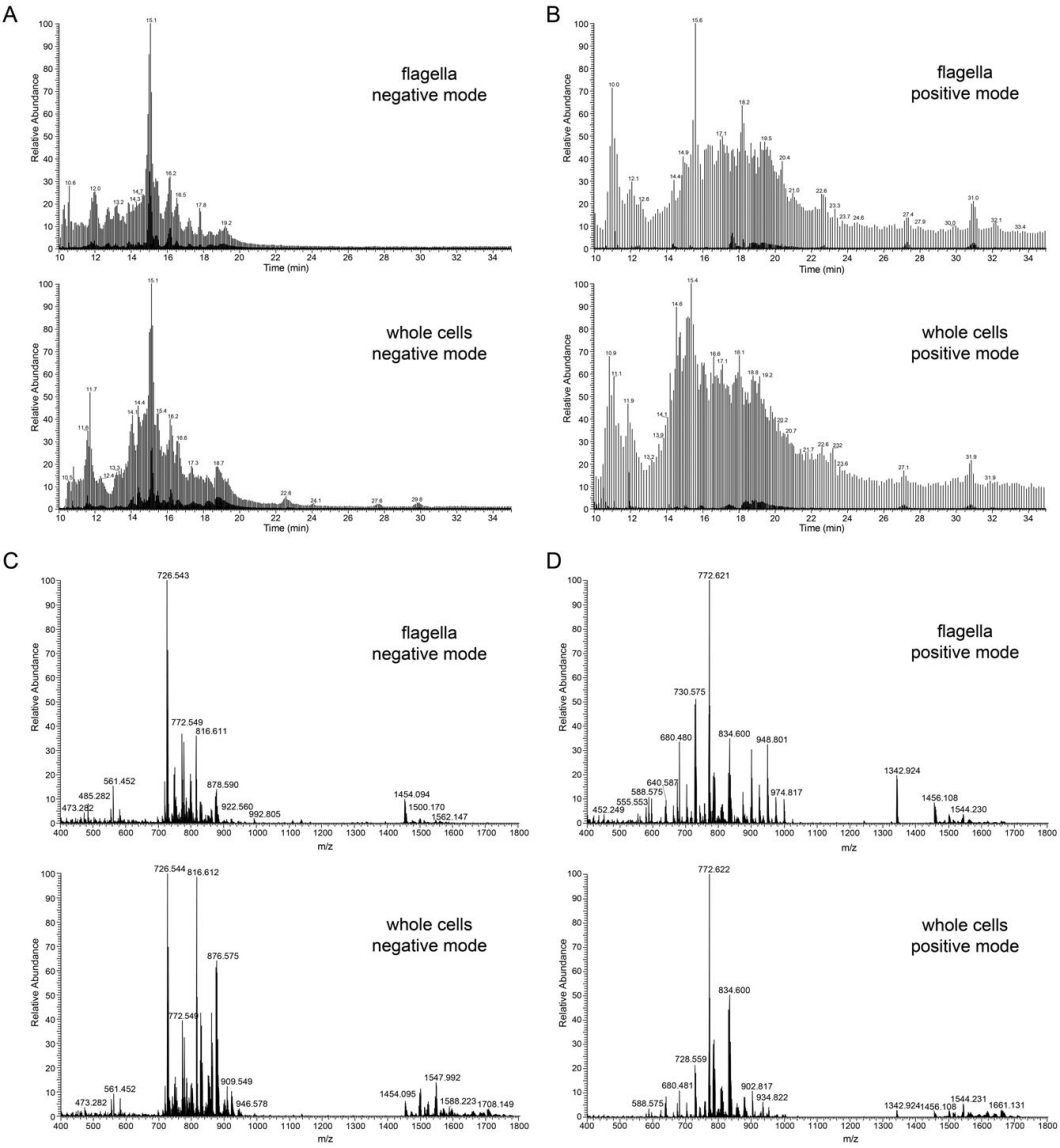
**Reverse-phase liquid chromatography separation and MS spectra.** (A,B) Total ion chromatograms of lipids extracted from isolated flagella (upper panels) and whole parasites (lower panels) acquired in negative (A) and positive (B) ionisation mode. Part of the chromatogram ranging from retention time 10 to 35 min is shown. (C,D) Sum spectra over the entire RT range in negative (C) and positive (D) mode of flagella (upper panels) and whole cells (lower panels).

**Fig. 3.**
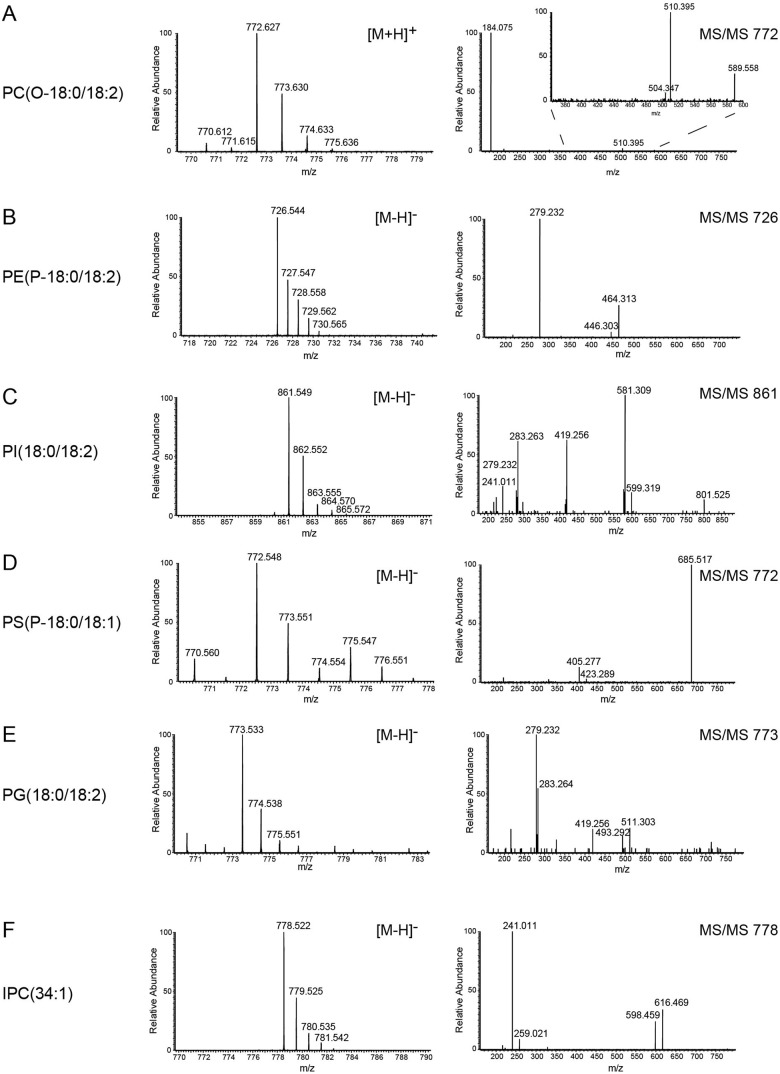
**Identification of phospholipid classes by MS.** Representative total ion spectra of the most intense species of PC (A), PE (B), PI (C), PS (D), PG (E) and IPC (F). Parent ion spectra in positive (PC) or negative (PE, PI, PS, PG and IPC) ionisation mode are shown in the left panel, and the corresponding MS/MS fragment spectra are depicted in the right panel.

**Fig. 4.**
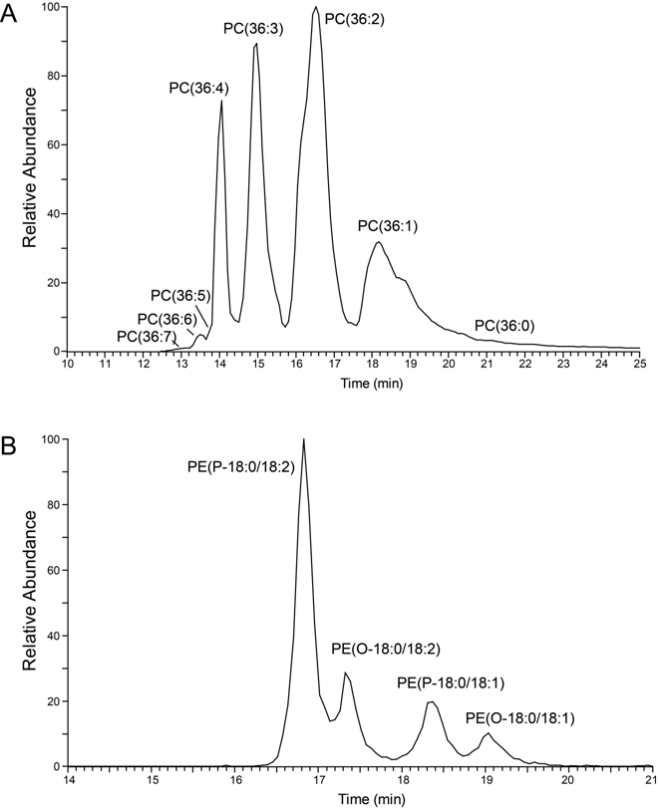
**Elution profiles of selected phospholipid molecular species.** (A) Elution profile of detected C_36_-PC molecular species in the positive ionisation mode of a whole cell lipid extract. (B) Elution profile of the most abundant ether-type PE molecular species detected in negative ionisation mode in whole cells. O refers to alkyl, P to alk-enyl molecular species.

The lipid composition of whole cells (containing all membranes, e.g. plasma membrane, endoplasmic reticulum, Golgi, nucleus, mitochondrion, glycosomes, acidocalcisomes) and purified flagella after tetracycline-induced down-regulation of Tb927.10.2880 was determined in two completely independent experiments. In each experiment, lipids were extracted and analysed by reverse-phase liquid chromatography high-resolution tandem mass spectrometry (LC-MS/MS) using a LTQ Orbitrap in both negative and positive ionisation modes. The separation of phospholipids by liquid chromatography was highly reproducible and allowed simultaneous acquisition of fragmentation spectra to facilitate identification of molecular species. Masses were acquired with a resolution of 60,000 with less than 1 ppm error, allowing identification of phospholipids based on their exact masses. Fig. 2A and B depict representative total ion chromatograms of lipid extracts of flagella (top panel) and whole cells (bottom panel) in negative (Fig. 2A) and positive (Fig. 2B) ionisation modes. Averaged total ion spectra across the retention time range 10–45 min from chromatograms in Fig. 2A and B are represented in Fig. 2C and D, respectively. Peak lists were extracted and subjected to several database searches (see Materials and Methods). Identification of individual phospholipid molecular species was based on collision-induced (CID) fragmentation using characteristic fragmentation patterns for the different phospholipid classes (Fig. 3). Using this LC-MS/MS protocol, we were able to identify a total of 216 phospholipid species in the negative and 193 species in the positive ionisation mode, with the majority of phospholipid classes and molecular species (>50%) confirmed by CID fragmentation. Two independent experiments were conducted, and only phospholipids identified in both experiments were considered real hits and used in our analyses. The resolution power of the liquid chromatography protocol is demonstrated in Fig. 4, showing the elution profiles of the major 36-carbon PC species in positive mode (panel A) and the major 36-carbon ether-type PE species in negative mode (panel B). The liquid chromatography method separated isobaric phospholipids with different types of linkages, allowing identification of alkyl-acyl versus alk-enyl-acyl (plasmalogen) molecular species (see Fig. 4B). We will first describe the complete set of lipids identified and then compare their relative abundances between whole cells and purified flagella.

### Identification of major phospholipid molecular species in flagella

#### Identification of PC molecular species

PC molecular species were identified in both negative and positive ionisation modes. In negative mode, formate adducts [M+CHO_2_]^−^ were detected, and collision-induced activation yielded [M-CH_3_-CHO_2_]^−^ ions, detected as neutral loss of 60 Da daughter fragments (see also Houjou et al., 2004; Kerwin et al., 1994), as well as low signals of free fatty acyl chains. In positive mode, PC molecular species were identified based on the characteristic phosphocholine daughter fragment at m/z 184 (Fig. 3A). In total, 77 distinct PC molecular species were identified in positive ionisation mode, of which 43 were also detected in negative mode (supplementary material Table S1). The most intense species detected in flagella in positive mode were m/z 772.621, m/z 770.606 and m/z 834.600, representing PC(O-18:0/18:2, with O referring to alkyl), PC(P-18:0/18:2, with P referring to alk-enyl) and PC(18:0/22:6), respectively. The same PC molecular species (O-18:0/18:2; P-18:0/18:2 and 18:0/22:6) were also the most intense peaks in negative mode, with m/z values of 816.611, 814.596 and 878.591, respectively (supplementary material Table S1). In contrast, in whole cell extracts diacyl-type PC molecular species were much more intense, with PC(18:0/22:6), PC(18:2/22:6) and PC(18:1/22:6) representing the most intense species, although PC(O-18:0/18:2) was still the most abundant PC peak. The identified PC molecular species are in good agreement with previous lipidomic analyses of whole cells of *T. brucei* procyclic forms (Patnaik et al., 1993; Richmond et al., 2010).

#### Identification of PE molecular species

Our analyses in negative mode revealed 32 PE molecular species, of which 13 belonged to ether-type subclasses, while in positive mode, 50 species were identified, with 17 representing ether-type PE molecular species (supplementary material Table S2). Individual species were confirmed by CID fragmentation involving fatty acid dissociation in negative mode (Fig. 3B), and by neutral loss of phosphoethanolamine (m/z 141 Da) in positive mode. In negative mode, the three most intense PE [M−H]^−^ parent ions in flagellar extracts were m/z 726.544 [PE(P-18:0/18:2)], m/z 728.559 [PE(P-18:0/18:1) and PE(O-18:0/18:2)] and m/z 730.575 [PE(O-18:0/18:1)]. These parent ions also represented the most abundant PE molecular species in positive mode. In addition, the same PE molecular species were also the most abundant ones in whole cell extracts. The identified PE molecular species are in good agreement with previous studies (Guler et al., 2008; Patnaik et al., 1993; Richmond et al., 2010; Signorell et al., 2008). As mentioned above, the liquid chromatography protocol was able to separate alkyl-acyl and alk-enyl-acyl (plasmalogen) molecular species having the same mass to charge value (see Fig. 4B).

#### Identification of PI molecular species

Analysis of PI revealed 38 different molecular species in negative mode as [M–H]^−^ ions in the range of m/z 800 to 960. PI species were identified based on the characteristic *myo*-inositol cyclic phosphate fragment of m/z 241 and the loss of individual fatty acyl anions (Fig. 3C). While the most prominent species were diacyl-type PIs with combined fatty acyl chain length and degree of unsaturation ranging from PI(32:0) to PI(42:10), we also identified three ether-type molecular species (supplementary material Table S3). The three most prominent PI molecular species detected in both flagella and whole cell lipid extracts were identified as PI(18:0/18:2), PI(18:1/18:1) and PI(18:0/18:1), with m/z values of 861.548 and 863.565 respectively. The PI molecular species identified are in good agreement with previous analyses of whole cells (Guler et al., 2008; Güther et al., 2006; Patnaik et al., 1993; Richmond et al., 2010).

#### Identification of PS molecular species

PS molecular species were detected in negative mode as [M−H]^−^ and in positive mode as [M+H]^+^ parent ions. Characteristic fragmentation resulting in neutral loss of the serine moiety C_3_H_5_NO_2_ (m/z 87) was used to identify PS species in negative mode (Fig. 3D), while neutral loss of phosphoserine (m/z 185) after CID was used to identify PS in positive mode (Brügger et al., 1997). The major PS molecular species were confirmed by MS3 analysis using direct infusion in negative mode. In total, we identified 47 and 25 PS molecular species in negative and positive mode, respectively (supplementary material Table S4). The most intense PS molecular species in both flagella and whole cells (in both positive and negative mode) were PS(P-18:1/18:0), PS(O-18:0/18:2), PS(O-18:0/18:1) and PS(P-20:0/18:1). Similar PS molecular species have been reported in whole cells in a previous study (Richmond et al., 2010).

#### Identification of phosphatidylglycerol (PG) and cardiolipin (CL) molecular species

PG molecular species can easily be detected in negative ionisation mode as [M-H]^−^ ions, and CID readily dissociates fatty acyl chains for easy molecular species determination (Fig. 3E). In total, we identified 16 PG molecular species, ranging from PG(34:1) to PG(44:12), with most species being of diacyl-type and only two having ether-type linkages (supplementary material Table S5). The most intense species detected in both flagella and whole cells represented PG(18:0/18:2) at m/z 773.533, PG(O-18:0/18:2) at m/z 759.554 and PG(18:0/18:1) at m/z 775.549. The PG molecular species identified are in good agreement with previous studies (Richmond et al., 2010; Serricchio and Bütikofer, 2013). CL was detected in negative and positive ionisation mode as [M−H]^−^ and [M+H]^+^, respectively. Several CL clusters in the range m/z 1500–1700 were identified in both flagella and whole cells (Fig. 2B). The most intense molecular species were detected in the cluster ranging from m/z 1454.09 to m/z 1462.16, representing CL(72:5) to CL(72:1). Unfortunately, no CID data for any of the CL species could be collected to allow determination of the molecular species composition. This is likely due to the use of collision energies that are unsuitable for efficient fragmentation of CL.

#### Identification of SM, IPC and ceramide (Cer) molecular species

SM molecular species were detected in negative mode as [M+CHO_2_]^−^ and in positive mode as [M+H]^+^ ions. Similar to PC, MS/MS analysis of formate adducts resulted in intense daughter ions after neutral loss of 60 Da, representing [M-CH_3_-CHO_2_]^−^ (Houjou et al., 2004; Kerwin et al., 1994). In positive mode, SM molecular species produced the characteristic fragment at m/z 184. In total, four distinct SM molecular species were detected in negative mode, while in positive mode eleven species could be identified (supplementary material Table S6). The most prominent species detected in flagella and whole cells in positive mode were SM(d32:1) at m/z 675.544, SM(d34:1) at m/z 703.575 and SM(d36:1) at m/z 731.606 (where d represents a dihydroxy long-chain base). The detailed structures of the backbones (dihydroceramide versus ceramide) of the SM species were not determined. In previous studies, *T. brucei* procyclic form SM species were reported to contain ceramide backbones (Richmond et al., 2010; Sutterwala et al., 2008). Based on that report, the species identified in our study likely represent SM(d18:1/14:0), SM(d18:1/16:0) and SM(d18:1/18:0).

IPC molecular species were detected in negative mode as [M−H]^−^ and in positive mode as [M+H]^+^ ions. All IPC species were confirmed by MS/MS analysis, which generated abundant daughter ions at m/z 241, representing loss of *myo*-inositol cyclic phosphate, and m/z 259, representing inositol monophosphate anion (Fig. 3F). In flagella, we identified 12 different species in negative mode, with IPC(34:1) at m/z 778.523, IPC(32:1) at m/z 750.492 and IPC(32:0) at m/z 752.509 representing the three most intense species (supplementary material Table S7). Similar *T. brucei* IPC molecular species have been reported previously (Fridberg et al., 2008; Guler et al., 2008; Güther et al., 2006; Serricchio and Bütikofer, 2013; Sutterwala et al., 2008). Mass to charge values corresponding to six different IPC species were identified in positive mode. The m/z values matched the species identified in negative mode. The detailed structures of the long chain bases were not determined. Based on an earlier analysis of *T. brucei* procyclic forms (Sutterwala et al., 2008), the IPC peaks listed above most likely represent IPC(d18:1/16:0), IPC(d16:0/16:1) and IPC(d16:0/16:0).

Finally, six different ceramide molecular species in the mass to charge range from 510 to 568 were identified in positive ionisation mode as [M+H]^+^ ions (supplementary material Table S8). The most intense species detected in flagella and whole cells were Cer(32:1) at m/z 510.488, Cer(34:1) at m/z 538.519 and Cer(36:1) at m/z 566.551. Some of these species have previously been described in *T. brucei* (Sutterwala et al., 2008). The detailed structures of the sphingoid bases of the Cer species were not determined.

### Relative changes in phospholipid composition between flagella and whole cells

**Fig. 5.**
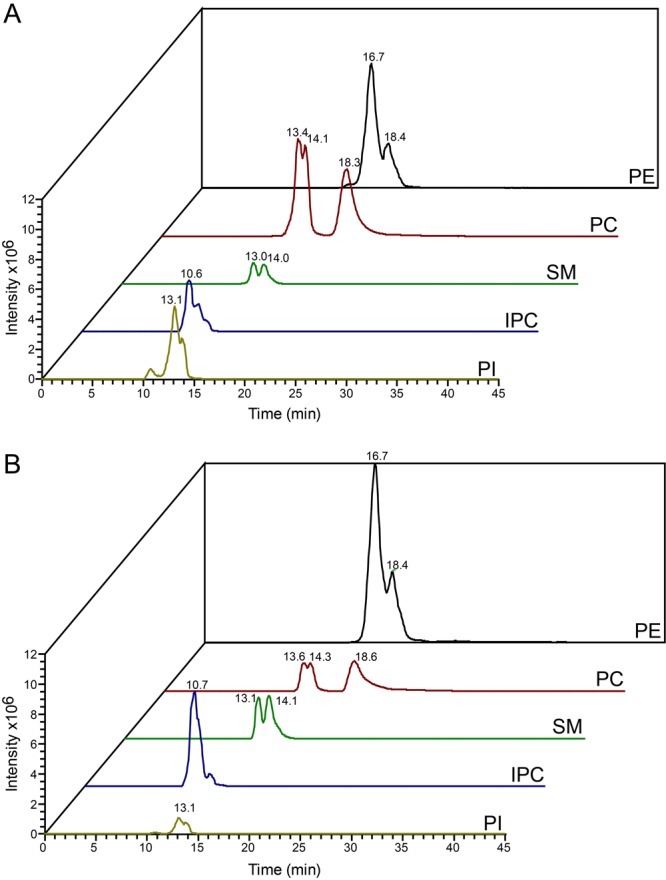
**Comparison of chromatograms of the most intense molecular species of different phospholipid classes acquired in negative ionisation mode.** Total ion chromatograms and retention times of the three most intense species of PE, PC, SM, IPC and PI of lipid extracts from whole cells (A) or isolated flagella (B).

**Fig. 6.**
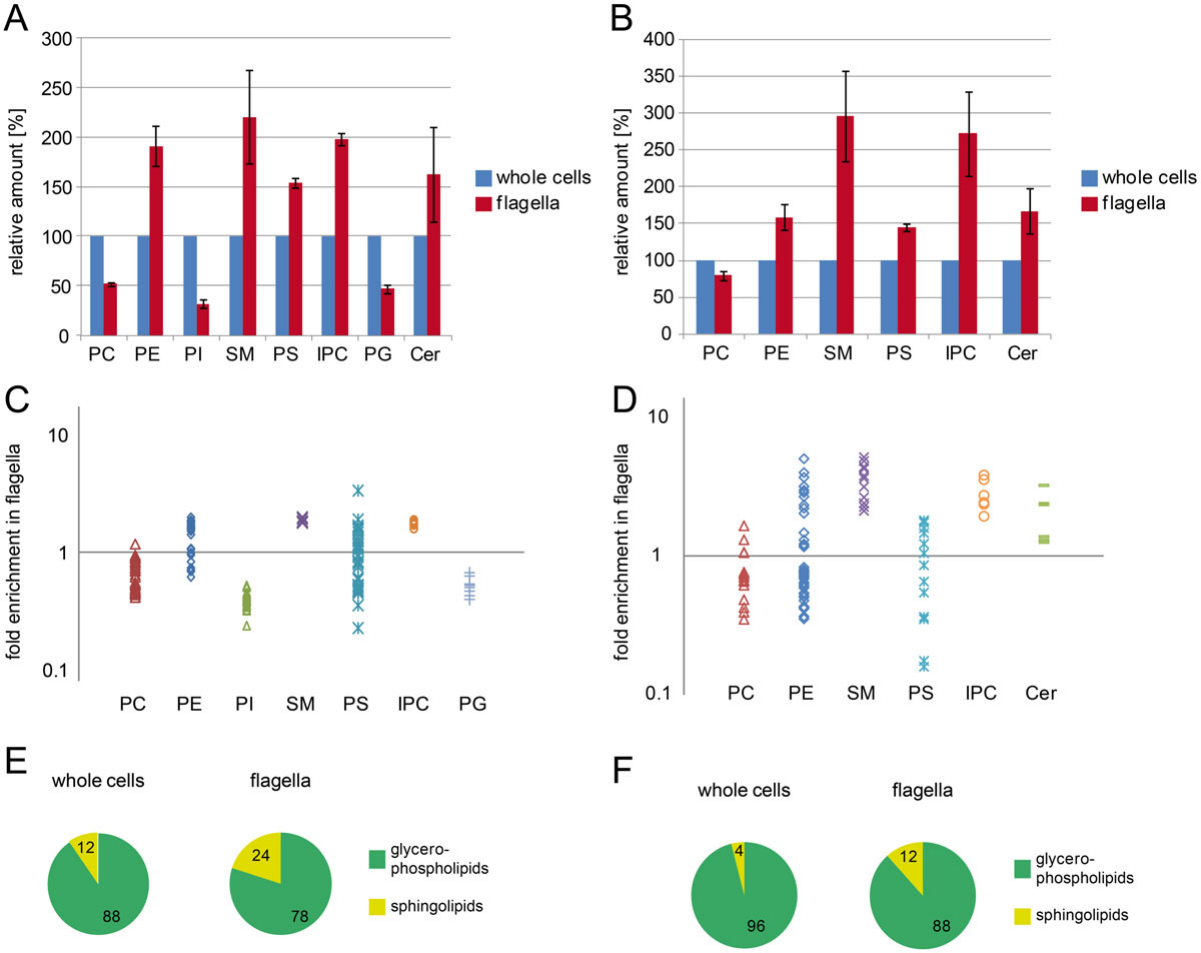
**Relative changes in phospholipid composition between whole cells and flagella.** (A,B) Mean signal intensities of two independent analyses of flagella relative to whole cells, obtained in negative ionisation mode (A) and positive ionisation mode (B) (mean values±s.e.m.). (C,D) Ratio (flagella to whole cells) of normalized signal intensities of each individual phospholipid detected in a given class in negative (C) and positive (D) ionisation mode. For clarity, species below a cut-off of 1% signal intensity relative to the most intense species were omitted. (E,F) Fraction of glycerophospholipids and sphingophospholipids detected in negative (E) or positive (F) ionisation mode represented as percentage of total lipids.

To determine depletion or enrichment of a phospholipid class in flagella relative to whole cells, the sums of the signal intensities of all molecular species of a given class were compared. To correct for intensity differences due to varying amounts of injected material between different runs, signal intensities of all identified phospholipids were summarized and normalized to match the corresponding reference sample. To visualize the relative changes between individual phospholipid classes, elution profiles of the three most intense species of PE, PC, SM, IPC and PI from whole cell extracts and flagella are depicted in Fig. 5. The comparison shows that PE, SM and IPC are highly enriched relative to PC and PI in flagella (Fig. 5B) compared to whole cells (Fig. 5A). Comparisons of the normalized signal intensities of all phospholipid classes (plus ceramide) between flagella and whole cells show that, independent of the ionisation mode used, PC, PI and PG were depleted in flagella by at least 45% (Fig. 6A,B), whereas PE, SM, PS, IPC and ceramide were enriched by 150–320% in flagella compared to whole cells (Fig. 6A,B). Analysis of molecular species within a given lipid class showed that nearly all species of PC, PI, SM, IPC, PG and ceramide behaved similarly, meaning that they were either enriched or depleted in flagella relative to whole cells (Fig. 6C,D). In contrast, among PE and PS molecular species some showed enrichment in flagella while others were depleted (Fig. 6C,D). Remarkably, we found that most ether-type PC molecular species were enriched in flagella relative to whole cells. The relative amounts of ether-type PC molecular species increased from 24.9±0.01% (analysis in negative mode) and 30.3±2.8% (analysis in positive mode) in whole cells to 37.5±2.2% and 44.9±2.9% in flagella, respectively (Table 1). A similar enrichment of ether-type molecular species in flagella versus whole cells was also observed for PS and PE (Table 1). Together these results demonstrate that ether-type phospholipid molecular species are clearly enriched in flagella compared to whole cells and that this relative change can be detected in both negative and positive mode of LC-MS/MS analysis.

**Table 1.**
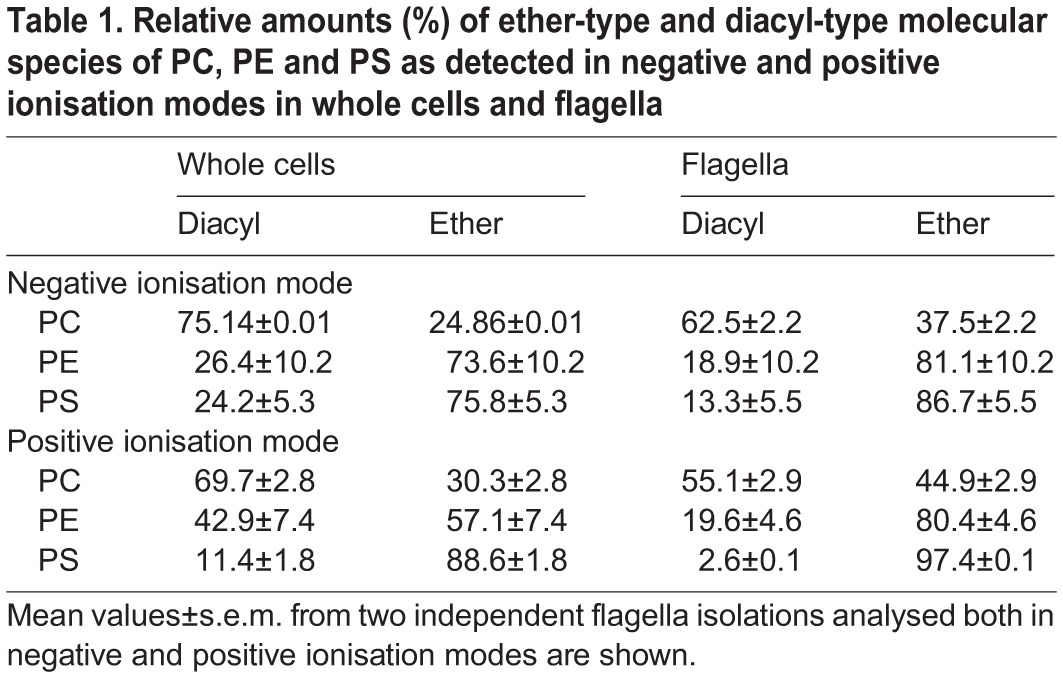
**Relative amounts (%) of ether-type and diacyl-type molecular species of PC, PE and PS as detected in negative and positive ionisation modes in whole cells and flagella**

In addition, we found a substantial enrichment of sphingolipid classes in flagella versus whole cells. The sum of the signal intensities of all sphingolipids increased from 12±3% (analysis in negative mode) and 4±1% (analysis in positive mode) in whole cells to 24±4% and 12±1% in flagella, respectively (Fig. 6E,F). The observed strong depletion (>50%) of the mitochondrial signature lipids, PG and CL (see Fig. 2C), in flagella compared to whole cells was expected, as the flagella preparation was devoid of cell remnants. On the other hand, the fact that residual amounts of PG and CL were detected in purified flagella can be explained by the close association of the mitochondrial membrane with the basal body of the flagellum (Ogbadoyi et al., 2003; Robinson and Gull, 1991).

**Fig. 7.**
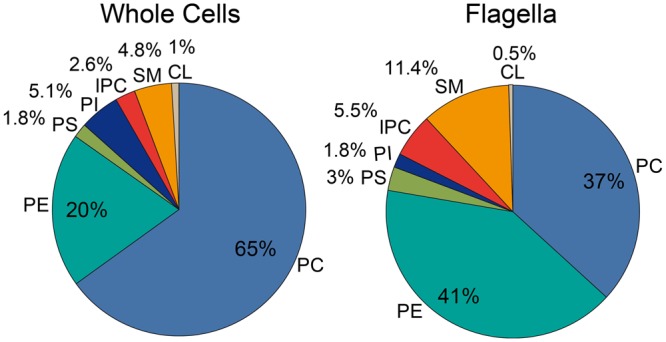
**Phospholipid composition of *T. brucei* parasites and flagella.** Left panel: The phospholipid composition of parasites was determined by two-dimensional thin-layer chromatography and lipid phosphorus quantification. The data are mean values from four separate determinations. Right panel: The phospholipid composition of flagella was calculated from the data shown in the left panel using the mean relative changes observed in two LC-MS analyses between flagella and whole cells.

The limited amounts of flagella available did not allow quantitative phospholipid analysis using lipid phosphorus determination. Nevertheless, based on our mass spectrometry data we were able to deduce the phospholipid composition of flagella membranes. For this we first determined the phospholipid composition of whole cells using two-dimensional thin layer chromatography followed by lipid phosphorus quantification (Fig. 7, left panel) and then calculated the phospholipid composition of flagella (Fig. 7, right panel) using the relative changes in individual phospholipid classes between whole cells and flagella determined by LC-MS analysis. The result shows that in contrast to whole cells, where PC is the most abundant phospholipid class, PE is the most abundant phospholipid class in flagella (increase from approximately 20% in whole cells to approximately 40% of total phospholipid in flagella). Accordingly, decreased relative amounts in flagella versus whole cells were observed for PC (37% vs 65%) and PI (2% vs 5%). In contrast, the relative amounts of PS, SM and IPC in flagella were increased compared to whole cells (3% vs 2%, 11% vs 5% and 6% vs 2%, respectively). Together, the relative amounts of the raft-typical sphingophospholipid classes, SM and IPC, were increased in flagella (17%) compared to whole cells (7%) (Fig. 7). These calculated numbers are in good agreement with the values determined by LC-MS/MS (Fig. 6E), where sphingophospholipid signal intensities accounted for 24% of total intensities in flagella and 12% in whole cells.

Formally, we cannot rule out that the lipid composition of *T. brucei* flagella determined in this study is identical to that of the plasma membrane. However, several studies have shown that the biophysical properties of the trypanosome flagellum are clearly different from the plasma membrane (see above). In addition, in eukaryotic cells where the plasma membrane lipid composition has been determined, PC was found to be the most abundant phospholipid class (Burger et al., 2007; Calderon and DeVries, 1997; Contreras et al., 1997; Malviya et al., 1986; Pankov et al., 2006). Since PC is by far the most abundant phospholipid class in *T. brucei* parasites (Fig. 7), it is likely that PC also represents the most abundant lipid in the trypanosome plasma membrane. In contrast, our result show that the most abundant phospholipid class in the *T. brucei* flagellum is PE, indicating that the lipid composition of the flagellum is different from that of the membrane of the remaining cell body. Thus, the phospholipid composition of flagella may represent a lipid environment specifically suited for the unique functions of a flagellum.

In summary, our molecular species analyses of flagella and whole cells demonstrate that PE, PS, IPC and SM, as well as Cer, are highly enriched in flagella compared to whole cells, whereas PC and PI are depleted. In addition, we found that within a given glycerophospholipid class, ether-type molecular species are enriched in flagella versus whole cells. These results indicate that flagella contain a lipid environment rich in sphingolipids and distinct glycerophospholipid molecular species, resembling raft-like microdomains of plasma membranes of eukaryotic cells (Brugger et al., 2006; Ostermeyer et al., 1999; Pike et al., 2002). Our interpretation that these membrane microdomains are bona fide raft membranes is in line with previous studies, in which the flagellar membrane was shown to be less fluid than the adjacent plasma membrane, possibly a result of enrichment of glycosphingolipids and sterols (De Souza, 1995; Tetley, 1986; Tyler et al., 2009). In addition, our data are in agreement with a study on isolated plasma membranes and lipid raft fractions of human epidermal carcinoma cells, in which electrospray-ionisation mass spectrometry revealed a depletion of PC and PI in raft membranes, whereas PS, PE and SM were enriched (Pike et al., 2002). Furthermore, the authors found a strong enrichment of ether-type (plasmanyl and plasmenyl) PE molecular species in lipid rafts (Pike et al., 2002), similar to our observations in *T. brucei* showing enrichment of ether-type molecular species of PE, PC and PS in flagella compared to whole cells. The lack of the carbonyl oxygen at the *sn1*-position renders ether-type phospholipids more lipophilic than corresponding diacyl molecular species. In addition, the orientation of the *sn2*-fatty acyl chain in ether-type glycerophospholipids is altered, making membranes containing these phospholipid sub-classes less fluid than membranes containing ester-type glycerophospholipids (reviewed in Paltauf, 1994). This biophysical behaviour may give ether-type lipids a higher propensity to organize in distinct membrane domains. A raft-like lipid composition, in turn, may promote enrichment (or depletion) of distinct proteins in flagella and influence their mobility and association with other membrane components (reviewed in Vincensini et al., 2011). In *T. brucei*, the proposed existence of a distinct lipid environment in *T. brucei* flagella was suggested to regulate trafficking of particles beneath the flagellar membrane (Tyler et al., 2009). Furthermore, it has been suggested that not only the flagellar membrane but also the flagellar pocket membrane of *T. brucei* parasites may be composed of distinct lipids, i.e. be enriched in sterols and depleted in sphingolipids (Fridberg et al., 2008), which may contribute to restricting movement of membrane components in and out of the flagellar pocket and on to the flagellum.

In conclusion, the present work represents the first detailed phospholipid analysis of flagella in any organism.

## MATERIALS AND METHODS

### Trypanosomes and culture conditions

*T. brucei* 29-13 procyclic forms co-expressing a T7 RNA polymerase and a tetracycline repressor (Wirtz et al., 1999) were cultured at 27°C in SDM-79 (Invitrogen, Basel, Switzerland) containing 10% (v/v) heat-inactivated fetal bovine serum (FBS), 25 µg/ml hygromycin and 15 µg/ml G418. The cell line expressing double-stranded RNA targeting gene Tb927.10.2880 was cultured in the same conditions but with the additional presence of 2 µg/ml puromycin.

### RNAi-mediated gene silencing

Expression of Tb927.10.2880 (annotated in GeneDB/TriTrypDB as calcium channel, putative) was down-regulated by RNAi using a stem-loop construct containing a puromycin resistance gene. Selection of the gene sequences for RNAi was done with RNAit (Redmond et al., 2003). A 692 bp fragment spanning nucleotides 548–1239 of Tb927.10.2880 was amplified by PCR using the forward primer 5′-GCCCAAGCTTGGATCCAATCTGTGGAGCAGCTATGT-3′ and the reverse primer 5′-CTGCTCTAGACTCGAGTATCATCAGCAAAAGCACTG-3′, and cloned into the target vector pALC14 (Bochud-Allemann and Schneider, 2002; a kind gift of A. Schneider, University of Bern). Before transfection, plasmid DNA was linearized with *Not*I and precipitated with phenol and chloroform.

### Stable transfection of trypanosomes and selection of clones

Parasites [4×10^7^ cells at mid-log growth phase (0.5–0.8×10^7^ cells/ml)] cells were harvested by centrifugation at 1250×***g*** for 10 min, washed once in buffer (132 mM NaCl, 8 mM KCl, 8 mM Na_2_HPO_4_, 1.5 mM KH_2_PO_4_, 0.5 mM magnesium acetate, 0.09 mM calcium acetate, pH 7.0), resuspended in 450 μl of the same buffer, and mixed with 15 μg of linearized plasmid. Electroporation was performed with a BTX Electroporation 600 System (Axon Lab, Baden, Switzerland) with one pulse (1.5 kV charging voltage, 2.5 kV resistance, 25 mF capacitance timing, and 186 Ω resistance timing) using a 0.2-cm pulse cuvette (Bio-Rad). Electroporated cells were immediately inoculated in 10 ml of SDM-79 containing 10% heat-inactivated FBS. Clones were obtained by limited dilution in 24-well plates in SDM-79, containing 20% conditioned medium, in the presence of 2 μg/ml puromycin for selection. Antibiotic-resistant clones were tested by PCR. RNAi was induced by addition of 1 μg/ml tetracycline to parasite cultures. Northern blot analysis was performed as previously described (Serricchio and Bütikofer, 2012) using a probe generated with primers described above.

### Flagella isolation, lipid extraction and quantification

To detach flagella from the cell body, RNAi against Tb927.10.2880 was induced by addition of tetracycline for 48 h and flagella from 4×10^9^ procyclic form trypanosomes were isolated as described before with minor modifications (Oberholzer et al., 2011; Subota et al., 2014). Parasites were washed and re-suspended in 4 ml of phosphate-buffered saline (PBS, 137 mM NaCl, 2.7 mM KCl, 10 mM Na_2_HPO_4_, 1.76 mM KH_2_PO_4_, pH 7.4), and passed 5 times through a 28 G needle. Cells were vortexed for 1 min and then layered on a 30% sucrose cushion. After centrifugation at 770×***g***, the top layer and interphase were removed and sucrose sedimentation was repeated. Flagella were collected by centrifugation at 16,000×***g*** for 30 min. The purity of the flagellar preparation was checked by differential interference contrast (DIC) and fluorescence microscopy using a Leica SP2 with a 100× oil objective. Flagella were mounted with Vectashield containing 4′,6-diamidino-2-phenylindol (DAPI). 10^8^ induced parasites before isolation of flagella were removed and used as control cells. Lipids of isolated flagella and control parasites were isolated according to the method of Bligh and Dyer (1959), dried under a stream of nitrogen and stored at −20°C until analysed. Two-dimensional thin-layer chromatography and lipid phosphorus quantification was done as described (Signorell et al., 2008). Four independent determinations using lipid extracts from 4×10^8^ parasites each were performed.

### Transmission electron microscopy

Flagella were stored in glycerol at −20°C and washed three times in 0.1 M cacodylate buffer and fixed for 10 min with 2.5% glutaraldehyde in the same buffer. Fixation was continued overnight with 2.5% glutaraldehyde and 4% paraformaldehyde. Pellets were washed three times in cacodylate buffer, treated with 1% osmium for 1 h 30 min at room temperature, washed three times in water and incubated for 1 h with 2% uranyl acetate (w/v) in 25% ethanol before grading series of dehydration in ethanol. Pellets were embedded in Epon and sectioned. Samples were contrasted with uranyl acetate (2%, w/v) for 45 min and with 80 mM lead citrate for 4 min. Observation was carried out using a Tecnai BioTWIN 120 cryo electron microscope.

### Immunofluorescence microscopy

Flagella stored in glycerol were washed twice in PBS to remove glycerol, then resuspended in PBS, spread on poly-L-Lysine coated slides and dried. Methanol fixation was performed for 5 min at −20°C, followed by rehydration for 15 min in PBS. Slides were incubated in humid chamber with two primary antibodies for 1 h at 37°C. An IgG1 monoclonal antibody against *T. brucei* calflagin (Giroud et al., 2009) was used as a membrane marker along with the axonemal marker mAb25 (Pradel et al., 2006) diluted 1/500 and 1/10 respectively in PBS-containing 0.1% (w/v) bovine serum albumin (BSA). Slides were washed 4 times for 5 min in PBS prior to incubation for 45 min at 37°C in humid chamber with the appropriate secondary antibodies coupled to Alexa Fluor 488 (anti-IgG1) and Alexa Fluor Cy3 (anti-IgG2a) diluted 1/400 in PBS containing 0.1% BSA. Following 3 additional washes for 5 min in PBS, 2 μg/ml DAPI dissolved in water was applied to slides for 1 min. After a last 5-min PBS wash, slides were mounted with a drop of Prolong gold anti-fading agent. Samples were visualized with a DMI4000 microscope (Leica) and images acquired with a CCD camera Orca 03G (Hamamatsu).

### LC-MS/MS analysis of lipid extracts

Dried lipid samples were resuspended in 50 μl chloroform/methanol (1/1, v/v) followed by 5 min sonication prior to injection. Samples (5 μl) were injected onto an Alltima C18, 3 μm, 50 mm×2.1 mm column (Sigma-Aldrich, Switzerland) equipped with an Ascentis Express C18 guard column and were eluted with an isocratic/step gradient flow (200 μl/min) of solvent B (10 mM ammonium formate in methanol) in solvent A (H_2_O/methanol, 95/5, with 10 mM ammonium formate). During injection solvent B was kept at 30% for 5 min followed by a step increase to 50% for 2 min and lipid elution at 100% of solvent B for an additional 38 min. All solvents were of HPLC grade and were purchased from Merck (Darmstadt, Germany). Ammonium formate (Fluka, Buchs, Switzerland) was of ≥99% analytical grade. High-resolution tandem mass spectrometry (MS/MS) was performed on a hybrid LTQ Orbitrap FTMS coupled with liquid chromatography (LC) using a LC-20ADXR Shimadzu HPLC pump. Ion detection was performed with a linear ion trap MS (LTQ XL MS, Thermo Scientific, Bremen, Germany). Lipid identification was achieved by collision-induced dissociation (CID) of singly charged (M−H)^−^ or (M+H)^+^ parent ions using a “Nth order double play” experimental method, alternating from full-scan to CID fragmentation (eV35) of the eight most intense peaks detected during a 45 min LC-gradient. MS spectra were acquired with 60,000 resolution for full-scans and 15,000 for fragment ions (MS/MS) respectively. Samples were analysed in positive and negative ion mode using following parameter settings for the HESI source: ion spray voltage, 3000 V (positive mode) and 2500 V (negative mode); ion source heater, 250°C; sheath gas, 10; auxiliary gas, 5; capillary temperature, 275°C.

### Infusion-MS analysis of lipid extracts

Lipid samples were infused at a flow rate of ∼250 nl/min using a TriVersa NanoMate robotic ESI source (Advion BioSystems) (gas pressure 0.40 psi; ionisation potential 1.3–1.6 kV) equipped with a standard ESI chip (Advion, Ithaca, NY). Ion detection was performed in positive and negative ionisation modes on a hybrid LTQ Orbitrap XL and lipid identification was achieved by CID and/or HCD (high-energy collisional dissociation) of singly charged parent ions.

### Data analysis

Mass spectrometry data was analysed manually using Qual Browser version 2.0.7 (Thermo Fischer Scientific Inc.). Peak lists of different retention time segments were extracted and possible hits of lipids were identified by applying tools of the lipid maps database (http://www.lipidmaps.org). Signal intensities of individual lipids were determined by averaging spectra during elution of the lipid from the column and normalizing to the total number of spectra of the run. Lipids were identified according to mass, and for most lipids fragmentation spectra were collected to confirm the molecular species. Only phospholipids identified in two identical independent experiments were considered for the analysis. To account for intensity differences due to varying amounts of injected material, signal intensities of all identified phospholipids were summarized and normalized to match the corresponding reference sample.

## Supplementary Material

Supplementary information
