# Supplementary material for: Flagellar membranes are rich in raft-forming phospholipids
Source: Biol Open. 2015 Aug 14;4(9):1143–53. doi: 10.1242/bio.011957 (PMC4582118; doi:10.1242/bio.011957)
Supplement: Supplementary information [file supp_4_9_1143__index.html]

Flagellar membranes are rich in raft-forming phospholipids — Supplementary information 

# Flagellar membranes are rich in raft-forming phospholipids

## BIO011957 Supplementary information

**Files in this Data Supplement:**

- Supplementary information
