# Supplementary material for: Flagellar membranes are rich in raft-forming phospholipids
Source: Biol Open. 2015 Aug 14;4(9):1143–53. doi: 10.1242/bio.011957 (PMC4582118; doi:10.1242/bio.011957)
Supplement: Supplementary information [file supp_bio.011957_BIO011957supp.pdf]

Supplementary Information:

Tables S1 – S8: Lipid molecular species identified in positive and negative ionisation modes. * d stands for diacyl-type, e for ether-type molecular species. Amounts are calculated relative to the most intense molecular species. C: number of carbon atoms in fatty acyl chains; DB: number of double bonds.

Table S1: PC, positive ionisation mode

Input Mass	Matched Mass	Δ	type*	C	DB	Species	Ion	% Flagella	% Whole cells
772.6217	772.6215	0.0002	e	36	2	PC(O-18:0/18:2)	[M+H]+	100	100
770.6064	770.6058	0.0006	e	36	2	PC(P-18:0/18:2)	[M+H]+	49	32
834.6001	834.6008	0.0007	d	40	6	PC(18:0/22:6)	[M+H]+	31	43
786.6010	786.6008	0.0002	d	36	2	PC(36:2)	[M+H]+	22	34
836.6149	836.6165	0.0016	d	40	5	PC(40:5)	[M+H]+	20	31
830.5697	830.5695	0.0002	d	40	8	PC(18:2/22:6)	[M+H]+	17	36
788.6161	788.6164	0.0003	d	36	1	PC(18:0/18:1)	[M+H]+	18	18
784.5855	784.5851	0.0004	d	36	3	PC(18:1/18:2)	[M+H]+	15	25
832.5842	832.5852	0.0010	d	40	7	PC(18:2/22:5)	[M+H]+	14	33
838.6316	838.6321	0.0005	d	40	4	PC(40:4)	[M+H]+	8	12
812.6163	812.6164	0.0001	d	38	3	PC(38:3)	[M+H]+	7	11
810.6009	810.6008	0.0001	d	38	4	PC(38:4)	[M+H]+	7	11
742.5749	742.5745	0.0004	e	34	2	PC(e34:2)	[M+H]+	7	4
840.6470	840.6477	0.0007	d	40	3	PC(40:3)	[M+H]+	7	10
808.5854	808.5852	0.0002	d	38	5	PC(38:5)	[M+H]+	5	7
814.6318	814.6321	0.0003	d	38	2	PC(38:2)	[M+H]+	5	7
774.6375	774.6371	0.0004	e	36	1	PC(e36:1)	[M+H]+	5	6
806.5696	806.5695	0.0001	d	38	6	PC(38:6)	[M+H]+	4	11
782.5697	782.5695	0.0002	d	36	4	PC(18:2/18:2)	[M+H]+	4	11
760.5853	760.5851	0.0002	d	34	1	PC(16:0/18:1)	[M+H]+	4	7
744.5907	744.5902	0.0005	e	34	1	PC(e34:1)	[M+H]+	4	4
758.5699	758.5695	0.0004	d	34	2	PC(16:1/18:1)	[M+H]+	3	6
798.6370	798.6371	0.0001	e	38	3	PC(e38:3)	[M+H]+	3	3
880.5861	880.5852	0.0009	d	44	11	PC(44:11)	[M+H]+	3	9
794.6030	794.6058	0.0028	e	38	4	PC(e38:4)	[M+H]+	4	3
800.6530	800.6528	0.0002	e	38	2	PC(e38:2)	[M+H]+	3	4
878.5697	878.5695	0.0002	d	44	12	PC(44:12)	[M+H]+	3	7
856.5848	856.5851	0.0003	d	42	9	PC(42:9)	[M+H]+	3	3
860.6161	860.6164	0.0003	d	42	7	PC(42:7)	[M+H]+	2	4
858.6005	858.6008	0.0003	d	42	8	PC(42:8)	[M+H]+	2	4
768.5905	768.5902	0.0003	e	36	4	PC(e36:4)	[M+H]+	2	2
816.6480	816.6477	0.0003	d	38	1	PC(38:1)	[M+H]+	2	2
862.6316	862.6321	0.0005	d	42	6	PC(42:6)	[M+H]+	2	3

Biology Open • Supplementary information

854.5691	854.5695	0.0004	d	42	10	PC(42:10)	[M+H]+	2	4
804.5543	804.5538	0.0005	d	38	7	PC(38:7)	[M+H]+	1	3
884.6160	884.6165	0.0005	d	44	9	PC(44:9)	[M+H]+	1	2
882.6024	882.6008	0.0016	d	44	10	PC(44:10)	[M+H]+	1	3
796.6213	796.6215	0.0002	e	38	4	PC(e38:4)	[M+H]+	1	1
716.5596	716.5589	0.0007	e	32	2	PC(e32:2)	[M+H]+	1	<1
756.5542	756.5538	0.0004	d	34	3	PC(34:3)	[M+H]+	1	2
746.6067	746.6058	0.0009	e	34	1	PC(e34:1)	[M+H]+	1	1
778.5385	778.5382	0.0003	d	36	6	PC(36:6)	[M+H]+	1	1
864.6470	864.6477	0.0007	d	42	5	PC(42:5)	[M+H]+	1	1
780.5543	780.5538	0.0005	d	36	5	PC(36:5)	[M+H]+	1	1
886.6319	886.6321	0.0002	d	44	8	PC(44:8)	[M+H]+	1	1
732.5541	732.5538	0.0003	d	32	1	PC(32:1)	[M+H]+	1	1
852.5536	852.5538	0.0002	d	42	11	PC(42:11)	[M+H]+	1	1
790.6327	790.6321	0.0006	d	36	0	PC(36:0)	[M+H]+	1	1
762.6018	762.6008	0.0010	d	34	0	PC(34:0)	[M+H]+	1	1
730.5384	730.5382	0.0002	d	32	2	PC(32:2)	[M+H]+	1	1
802.5384	802.5382	0.0002	d	38	8	PC(38:8)	[M+H]+	<1	2
842.6639	842.6633	0.0006	d	40	2	PC(40:2)	[M+H]+	<1	1
734.5700	734.5695	0.0005	d	32	0	PC(32:0)	[M+H]+	<1	1
820.6222	820.6215	0.0007	e	40	6	PC(e40:6)	[M+H]+	<1	<1
866.6637	866.6634	0.0003	d	42	4	PC(42:4)	[M+H]+	<1	1
718.5752	718.5745	0.0007	e	32	1	PC(e32:1)	[M+H]+	<1	<1
754.5384	754.5382	0.0002	d	34	4	PC(34:4)	[M+H]+	<1	1
828.5537	828.5538	0.0001	d	40	9	PC(40:9)	[M+H]+	<1	1
888.6479	888.6478	0.0001	d	44	7	PC(44:7)	[M+H]+	<1	<1
844.6795	844.6790	0.0005	d	40	1	PC(40:1)	[M+H]+	<1	<1
766.5741	766.5745	0.0004	e	36	5	PC(e36:5)	[M+H]+	<1	<1
764.5578	764.5589	0.0011	e	36	6	PC(36:6)	[M+H]+	<1	<1
890.6636	890.6634	0.0002	d	44	6	PC(44:6)	[M+H]+	<1	<1
822.6373	822.6371	0.0002	e	40	5	PC(40:5)	[M+H]+	<1	<1
868.6793	868.6790	0.0003	d	42	3	PC(42:3)	[M+H]+	<1	<1
706.5383	706.5382	0.0001	d	30	0	PC(30:0)	[M+H]+	<1	<1
728.5232	728.5225	0.0007	d	32	3	PC(32:3)	[M+H]+	<1	<1
826.5373	826.5382	0.0009	d	40	10	PC(40:10)	[M+H]+	<1	<1
824.6536	824.6528	0.0008	e	40	4	PC(e40:4)	[M+H]+	<1	<1
826.6686	826.6684	0.0002	e	40	3	PC(e40:3)	[M+H]+	<1	<1
704.5226	704.5225	0.0001	d	30	1	PC(30:1)	[M+H]+	<1	<1
848.6529	848.6528	0.0001	e	42	6	PC(e42:6)	[M+H]+	<1	<1
750.5073	750.5069	0.0004	d	34	6	PC(34:6)	[M+H]+	<1	<1
702.5069	702.5069	0.0000	d	30	2	PC(30:2)	[M+H]+	<1	<1
776.5226	776.5225	0.0001	d	36	7	PC(36:7)	[M+H]+	<1	<1
892.6799	892.679	0.0009	d	44	5	PC(44:5)	[M+H]+	<1	<1
870.6958	870.6947	0.0011	d	42	2	PC(42:2)	[M+H]+	<1	<1

Biology Open • Supplementary information

PC, negative ionisation mode

Input Mass	Matched Mass	Δ	type*	C	DB	Species	Ion	% Flagella	% Whole cells
816.6114	816.6119	0.0005	e	36	2	PC(O-18:0/18:2)	[M+Formate]-	100	100
814.5963	814.5963	0.0000	e	36	2	PC(P-18:0/18:2)	[M+Formate]-	56	38
878.5909	878.5912	0.0003	d	40	6	PC(18:0/22:6)	[M+Formate]-	39	58
876.5749	876.5755	0.0006	d	40	7	PC(18:2/22:5)	[M+Formate]-	34	60
874.5593	874.5599	0.0006	d	40	8	PC(18:2/22:6)	[M+Formate]-	32	57
830.5909	830.5911	0.0002	d	36	2	PC(36:2)	[M+Formate]-	29	39
828.5755	828.5755	0.0000	d	36	3	PC(18:1/18:2)	[M+Formate]-	24	37
802.5602	802.5599	0.0003	d	34	2	PC(16:1/18:1)	[M+Formate]-	23	18
880.6063	880.6068	0.0005	d	40	5	PC(40:5)	[M+Formate]-	23	32
832.6059	832.6068	0.0009	d	36	1	PC(18:0/18:1)	[M+Formate]-	18	18
850.5603	850.5599	0.0004	d	38	6	PC(38:6)	[M+Formate]-	10	19
826.5601	826.5603	0.0002	d	36	4	PC(18:2/18:2)	[M+Formate]-	10	20
852.5761	852.5755	0.0006	d	38	5	PC(38:5)	[M+Formate]-	9	17
856.6065	856.6068	0.0003	d	38	3	PC(38:3)	[M+Formate]-	9	13
854.5913	854.5912	0.0001	d	38	4	PC(38:4)	[M+Formate]-	8	14
804.5751	804.5755	0.0004	d	34	1	PC(16:0/18:1)	[M+Formate]-	8	9
882.6217	882.6225	0.0008	d	40	4	PC(40:4)	[M+Formate]-	8	11
922.5591	922.5599	0.0008	d	44	12	PC(44:12)	[M+Formate]-	7	11
884.6380	884.6381	0.0001	d	40	3	PC(40:3)	[M+Formate]-	7	7
818.6281	818.6276	0.0005	e	36	0	PC(e36:0)	[M+Formate]-	7	5
788.5807	788.5811	0.0004	e	34	1	PC(e34:1)	[M+Formate]-	7	6
844.6434	844.6432	0.0002	e	38	2	PC(e38:2)	[M+Formate]-	6	4
924.5751	924.5756	0.0005	d	44	11	PC(44:11)	[M+Formate]-	6	10
858.6223	858.6225	0.0002	d	38	2	PC(38:2)	[M+Formate]-	5	6
900.5754	900.5755	0.0001	d	42	9	PC(42:9)	[M+Formate]-	4	8
798.5281	798.5286	0.0005	d	34	4	PC(34:4)	[M+Formate]-	4	3
844.6062	844.6068	0.0006	d	37	2	PC(37:2)	[M+Formate]-	3	2
902.5908	902.5912	0.0004	d	42	8	PC(42:8)	[M+Formate]-	3	6
898.5592	898.5599	0.0007	d	42	10	PC(42:10)	[M+Formate]-	3	6
848.5444	848.5442	0.0002	d	38	7	PC(38:7)	[M+Formate]-	3	5
822.5265	822.5286	0.0021	d	36	6	PC(36:6)	[M+Formate]-	3	2
926.5906	926.5912	0.0006	d	44	10	PC(44:10)	[M+Formate]-	3	4
904.6063	904.6068	0.0005	d	42	7	PC(42:7)	[M+Formate]-	2	4
928.6063	928.6069	0.0006	d	44	9	PC(44:9)	[M+Formate]-	2	3
906.6218	906.6225	0.0007	d	42	6	PC(42:6)	[M+Formate]-	2	3
846.6224	846.6225	0.0001	d	37	1	PC(37:1)	[M+Formate]-	1	1
824.5433	824.5442	0.0009	d	36	5	PC(36:5)	[M+Formate]-	1	2
886.6541	886.6538	0.0003	d	40	2	PC(40:2)	[M+Formate]-	1	1
776.5443	776.5442	0.0001	d	32	1	PC(32:1)	[M+Formate]-	1	1
908.6387	908.6382	0.0005	d	42	5	PC(42:5)	[M+Formate]-	1	1
888.6697	888.6694	0.0003	d	40	1	PC(40:1)	[M+Formate]-	<1	<1
790.5966	790.5967	0.0001	e	36	1	PC(O-16:0/18:1)	[M+Formate]-	<1	<1
910.6540	910.6538	0.0002	d	42	4	PC(42:4)	[M+Formate]-	<1	<1

Biology Open • Supplementary information

Table S2: PE, positive ionisation mode

Input Mass	Matched Mass	Δ	type*	C	DB	Species	Ion	% Flagella	% Whole cells
730.5750	730.5745	0.0005	e	36	2	PE(O-18:0/18:2);(P-18:0/18:1)	[M+H]+	100	83
728.5594	728.5589	0.0005	e	36	2	PE(P-18:0/18:2)	[M+H]+	99	100
732.5904	732.5902	0.0002	e	36	1	PE(O-18:0/18:1)	[M+H]+	32	26
758.6066	758.6058	0.0008	e	38	1	PE(O-20:0/18:2);(P-20:0/18:1)	[M+H]+	16	26
756.5909	756.5902	0.0007	e	38	3	PE(P-20:0/18:2)	[M+H]+	16	17
744.5544	744.5538	0.0006	d	36	2	PE(36:2)	[M+H]+	6	10
786.6371	786.6371	0.0000	e	40	2	PE(e40:2)	[M+H]+	5	13
772.5856	772.5851	0.0005	d	38	2	PE(38:2)	[M+H]+	4	13
714.5437	714.5432	0.0005	e	35	3	PE(e35:3)	[M+H]+	4	4
746.5701	746.5695	0.0006	d	36	1	PE(36:1)	[M+H]+	4	6
798.6011	798.6008	0.0003	d	40	3	PE(40:3)	[M+H]+	4	15
800.6157	800.6164	0.0007	d	40	2	PE(40:2)	[M+H]+	4	10
752.5574	752.5589	0.0015	e	38	5	PE(e38:5)	[M+H]+	3	2
784.6214	784.6215	0.0001	e	40	3	PE(e40:3)	[M+H]+	3	5
820.5857	820.5852	0.0005	d	42	6	PE(42:6)	[M+H]+	3	11
774.6006	774.6008	0.0002	d	38	1	PE(38:1)	[M+H]+	3	7
750.5410	750.5432	0.0022	e	38	6	PE(e38:6)	[M+H]+	3	2
822.6014	822.6008	0.0006	d	42	5	PE(42:5)	[M+H]+	2	9
760.6213	760.6215	0.0002	e	38	1	PE(e38:1)	[M+H]+	2	3
726.5430	726.5432	0.0002	e	36	4	PE(e36:4)	[M+H]+	2	2
754.5746	754.5745	0.0001	e	38	4	PE(e38:4)	[M+H]+	2	2
802.6321	802.6321	0.0000	d	40	1	PE(40:1)	[M+H]+	2	6
700.5279	700.5276	0.0003	e	34	3	PE(e34:3)	[M+H]+	2	2
702.5437	702.5432	0.0005	e	34	2	PE(e34:2)	[M+H]+	2	1
792.5542	792.5539	0.0003	d	40	6	PE(40:6)	[M+H]+	2	4
770.5697	770.5695	0.0002	d	38	3	PE(38:3)	[M+H]+	2	7
796.5854	796.5851	0.0003	d	40	4	PE(40:4)	[M+H]+	1	6
824.6170	824.6164	0.0006	d	42	4	PE(42:4)	[M+H]+	1	6
826.6322	826.6321	0.0001	d	42	3	PE(42:3)	[M+H]+	1	4
794.5693	794.5695	0.0002	d	40	5	PE(40:5)	[M+H]+	1	4
818.5702	818.5695	0.0007	d	42	7	PE(42:7)	[M+H]+	1	6
846.6004	846.6008	0.0004	d	44	7	PE(44:7)	[M+H]+	1	5
742.5386	742.5382	0.0004	d	36	3	PE(36:3)	[M+H]+	1	4
848.6161	848.6164	0.0003	d	44	6	PE(44:6)	[M+H]+	1	4
768.5541	768.5538	0.0003	d	38	4	PE(38:4)	[M+H]+	1	3
718.5387	718.5382	0.0005	d	34	1	PE(34:1)	[M+H]+	1	1
724.5279	724.5276	0.0003	e	36	5	PE(e36:5)	[M+H]+	1	4
740.5229	740.5225	0.0004	d	36	4	PE(36:4)	[M+H]+	1	2
828.6481	828.6477	0.0004	d	42	2	PE(42:2)	[M+H]+	1	2
850.6322	850.6321	0.0001	d	44	5	PE(44:5)	[M+H]+	1	2
816.5543	816.5539	0.0004	d	42	8	PE(42:8)	[M+H]+	1	4
788.5231	788.5226	0.0005	d	40	8	PE(40:8)	[M+H]+	<1	1

780.5881	780.5902	0.0021	e	40	5	PE(e40:5)	[M+H]+	<1	<1
790.5387	790.5382	0.0005	d	40	7	PE(40:7)	[M+H]+	<1	2
716.5225	716.5225	0.0000	d	34	2	PE(34:2)	[M+H]+	<1	1
766.5383	766.5382	0.0001	d	38	5	PE(38:5)	[M+H]+	<1	1
852.6478	852.6477	0.0001	d	44	4	PE(44:4)	[M+H]+	<1	1
844.5846	844.5851	0.0005	d	44	8	PE(44:8)	[M+H]+	<1	2
764.5229	764.5225	0.0004	d	38	6	PE(38:6)	[M+H]+	<1	1
854.6632	854.6634	0.0002	d	44	3	PE(44:3)	[M+H]+	<1	<1
688.4914	688.4912	0.0002	d	32	2	PE(32:2)	[M+H]+	<1	<1
714.5075	714.5069	0.0006	d	34	3	PE(34:3)	[M+H]+	<1	<1
690.5073	690.5069	0.0004	d	32	1	PE(32:1)	[M+H]+	<1	<1
842.5688	842.5695	0.0007	d	44	9	PE(44:9)	[M+H]+	<1	<1
762.5070	762.5069	0.0001	d	38	7	PE(38:7)	[M+H]+	<1	<1
712.4917	712.4912	0.0005	d	34	4	PE(34:4)	[M+H]+	<1	<1

Table S2: PE, negative ionisation mode

Input Mass	Matched Mass	Δ	type*	C	DB	Species	Ion	% Flagella	% Whole cells
726.5431	726.5443	0.0012	e	36	2	PE(P-18:0/18:2)	[M-H]-	100	100
728.5587	728.5599	0.0012	e	36	1	PE(O-18:0/18:2);PE(P-18:0/18:1)	[M-H]-	69	66
730.5752	730.5756	0.0004	e	36	1	PE(O-18:0/18:1)	[M-H]-	16	15
754.5749	754.5756	0.0007	e	38	3	PE(P-20:0/18:2)	[M-H]-	8	9
756.5910	756.5912	0.0002	e	38	2	PE(O-20:0/18:2);(P-20:0/18:1)	[M-H]-	7	6
742.5385	742.5392	0.0007	d	36	2	PE(18:0/18:2);PE(18:1/18:1)	[M-H]-	4	8
744.5538	744.5549	0.0011	d	36	1	PE(18:0/18:1)	[M-H]-	3	5
712.5282	712.5286	0.0004	e	35	2	PE(P-18:0/17:2)	[M-H]-	3	3
698.5124	698.5130	0.0006	e	34	3	PE(P-16:1/18:2)	[M-H]-	3	2
768.5547	768.5549	0.0002	d	38	3	PE(38:3)	[M-H]-	2	2
758.6068	758.6069	0.0001	e	38	1	PE(O-20:0/18:1)	[M-H]-	2	1
724.5281	724.5286	0.0005	e	36	3	PE(e36:3)	[M-H]-	2	2
714.5441	714.5443	0.0002	e	35	1	PE(P-18:0/17:1)	[M-H]-	2	2
740.5232	740.5236	0.0004	d	36	3	PE(18:1/18:2)	[M-H]-	2	5
770.5705	770.5705	0.0000	d	38	2	PE(18:0/20:2);PE(18:1/20:1)	[M-H]-	2	2
700.5282	700.5286	0.0004	e	34	1	PE(P-16:0/18:1)	[M-H]-	2	1
790.5388	790.5392	0.0004	d	40	6	PE(18:0/22:6)	[M-H]-	1	3
752.5590	752.5599	0.0009	e	38	4	PE(P-20:1/18:2)	[M-H]-	1	1
788.5237	788.5236	0.0001	d	40	7	PE(18:2/22:5);PE(18:1/22:6)	[M-H]-	1	2
792.5542	792.5549	0.0007	d	40	5	PE(18:0/22:5)	[M-H]-	1	2
716.5231	716.5236	0.0005	d	34	1	PE(16:0/18:1)	[M-H]-	1	1
738.5072	738.5079	0.0007	d	36	4	PE(18:2/18:2)	[M-H]-	1	3
796.5855	796.5862	0.0007	d	40	3	PE(40:3)	[M-H]-	1	1
714.5076	714.5079	0.0003	d	34	2	PE(16:0/18:2)	[M-H]-	1	1
786.5070	786.5079	0.0009	d	40	8	PE(40:8)	[M-H]-	<1	2

794.5703	794.5705	0.0002	d	40	4	PE(40:4)	[M-H]-	<1	1
750.5432	750.5443	0.0011	e	38	5	PE(e38:5)	[M-H]-	<1	<1
766.5389	766.5392	0.0003	d	38	4	PE(38:4)	[M-H]-	<1	1
772.5862	772.5862	0.0030	d	38	1	PE(38:1)	[M-H]-	<1	<1
702.5434	702.5443	0.0009	e	34	1	PE(e34:1)	[M-H]-	<1	<1
686.4753	686.4766	0.0013	d	32	2	PE(32:2)	[M-H]-	<1	<1
674.5128	674.5130	0.0002	e	32	0	PE(e32:1)	[M-H]-	<1	<1
712.4912	712.4923	0.0011	d	34	3	PE(34:3)	[M-H]-	<1	<1
798.6008	798.6018	0.0010	d	40	2	PE(40:2)	[M-H]-	<1	<1

Biology Open • Supplementary information

Table S3: PI, negative ionisation mode

Input Mass	Matched Mass	Δ	type*	C	DB	Species	Ion	% Flagella	% Whole cells
861.5489	861.5498	0.0009	d	36	2	PI(18:0/18:2);(18:1/18:1)	[M-H]-	100	100
863.5651	863.5655	0.0004	d	36	1	PI(18:0/18:1)	[M-H]-	91	67
909.5490	909.5498	0.0008	d	40	6	PI(18:0/22:6)	[M-H]-	28	29
859.5335	859.5342	0.0007	d	36	3	PI(18:2/18:1)	[M-H]-	18	21
911.5652	911.5655	0.0003	d	40	5	PI(18:0/22:5)	[M-H]-	13	11
835.5340	835.5342	0.0002	d	34	1	PI(16:0/18:1)	[M-H]-	11	11
833.5178	833.5185	0.0007	d	34	2	PI(16:0/18:2)	[M-H]-	10	14
847.5703	847.5706	0.0003	e	36	2	PI(O-18:0/18:2)	[M-H]-	9	6
907.5340	907.5342	0.0002	d	40	7	PI(18:1/22:6)	[M-H]-	7	7
847.5328	847.5342	0.0014	d	35	2	PI(35:2)	[M-H]-	7	8
885.5487	885.5498	0.0011	d	38	4	PI(18:0/20:4)	[M-H]-	5	5
887.5649	887.5655	0.0006	d	38	3	PI(38:3)	[M-H]-	4	3
857.5178	857.5185	0.0007	d	36	4	PI(18:2/18:2)	[M-H]-	4	4
905.5178	905.5185	0.0007	d	40	8	PI(18:2/22:6)	[M-H]-	4	4
913.5807	913.5811	0.0004	d	40	4	PI(40:4)	[M-H]-	3	3
889.5803	889.5811	0.0008	d	38	2	PI(38:2)	[M-H]-	3	3
915.5961	915.5968	0.0007	d	40	3	PI(40:3)	[M-H]-	3	3
883.5341	883.5342	0.0001	d	38	5	PI(38:5)	[M-H]-	2	3
881.5180	881.5185	0.0005	d	38	6	PI(38:6)	[M-H]-	2	3
849.5857	849.5862	0.0005	e	36	1	PI(e36:1)	[M-H]-	1	1
831.5024	831.5029	0.0005	d	34	3	PI(34:3)	[M-H]-	1	2
805.4861	805.4872	0.0011	d	32	2	PI(32:2)	[M-H]-	1	<1
931.5333	931.5342	0.0009	d	42	9	PI(42:9)	[M-H]-	<1	1
845.5181	845.5185	0.0004	d	35	3	PI(35:3)	[M-H]-	<1	1
933.5483	933.5498	0.0015	d	42	8	PI(42:8)	[M-H]-	<1	1
845.5553	845.5549	0.0004	e	36	3	PI(e36:3)	[M-H]-	<1	1
953.5184	953.5185	0.0001	d	44	12	PI(44:12)	[M-H]-	<1	1
955.5338	955.5340	0.0002	d	44	11	PI(44:11)	[M-H]-	<1	1
935.5645	935.5655	0.0010	d	42	7	PI(42:7)	[M-H]-	<1	<1
807.5025	807.5029	0.0004	d	32	1	PI(32:1)	[M-H]-	<1	1
809.5176	809.5185	0.0009	d	32	0	PI(32:0)	[M-H]-	<1	<1
929.5195	929.5186	0.0009	d	42	10	PI(42:10)	[M-H]-	<1	1
959.5646	959.5654	0.0008	d	44	9	PI(44:9)	[M-H]-	<1	<1
937.5802	937.5811	0.0009	d	42	6	PI(42:6)	[M-H]-	<1	<1
957.5491	957.5498	0.0007	d	44	10	PI(44:10	[M-H]-	<1	<1
879.5026	879.5029	0.0003	d	38	7	PI(38:7)	[M-H]-	<1	<1
829.4867	829.4872	0.0005	d	34	4	PI(34:4)	[M-H]-	<1	<1
917.6112	917.6124	0.0012	d	40	2	PI(40:2)	[M-H]-	<1	<1

Biology Open • Supplementary information

Table S4: PS, positive ionisation mode

Input Mass	Matched Mass	Δ	type*	C	DB	Species	Ion	% Flagella	% Whole cells
774.5649	774.5644	0.0005	e	36	2	PS(e36:2)	[M+H]+	100	100
776.5811	776.5800	0.0011	e	36	1	PS(e36:1)	[M+H]+	35	35
802.5960	802.5957	0.0003	e	38	2	PS(e38:2)	[M+H]+	23	27
772.5486	772.5487	0.0001	e	36	3	PS(e36:3)	[M+H]+	11	11
804.6114	804.6113	0.0001	e	38	1	PS(e38:1)	[M+H]+	8	9
824.5802	824.5801	0.0001	e	40	5	PS(e40:5)	[M+H]+	6	2
822.5645	822.5644	0.0001	e	40	6	PS(e40:6)	[M+H]+	3	4
826.5966	826.5957	0.0009	e	40	4	PS(e40:4)	[M+H]+	3	1
792.5753	792.5749	0.0004	d	36	0	PS(36:0)	[M+H]+	2	11
778.5963	778.5957	0.0006	e	36	0	PS(e36:0)	[M+H]+	2	4
838.5602	838.5593	0.0009	d	40	5	PS(40:5)	[M+H]+	2	4
852.6126	852.6114	0.0012	e	42	5	PS(e42:5)	[M+H]+	1	3
840.5755	840.5749	0.0006	d	40	4	PS(40:4)	[M+H]+	1	4
828.6120	828.6113	0.0007	e	40	3	PS(e40:3)	[M+H]+	1	1
848.5806	848.5800	0.0006	e	42	7	PS(P-20:0/22:6)	[M+H]+	1	8
760.5492	760.5487	0.0005	e	35	2	PS(e35:2)	[M+H]+	1	<1
850.5968	850.5957	0.0011	e	42	6	PS(e42:6)	[M+H]+	1	6
762.5274	762.5280	0.0006	d	34	1	PS(34:1)	[M+H]+	1	1
864.5752	864.5750	0.0002	d	42	6	PS(42:6)	[M+H]+	1	6
842.5911	842.5906	0.0005	d	40	3	PS(40:3)	[M+H]+	<1	2
866.5917	866.5906	0.0011	d	42	5	PS(42:5)	[M+H]+	<1	2
836.5439	836.5437	0.0002	d	40	6	PS(40:6)	[M+H]+	<1	1
862.5596	862.5593	0.0003	d	42	7	PS(42:7)	[M+H]+	<1	6
814.5597	814.5593	0.0004	d	38	3	PS(38:3)	[M+H]+	<1	3
762.5638	762.5644	0.0006	e	35	1	PS(e35:1)	[M+H]+	<1	<1

PS, negative ionisation mode

Input Mass	Matched Mass	Δ	type*	C	DB	Species	Ion	% Flagella	% Whole cells
772.5481	772.5498	0.0017	e	36	2	PS(P-18:0/18:1)	[M-H]-	100	100
798.5651	798.5654	0.0003	e	38	3	PS(e38:3)	[M-H]-	54	32
774.5655	774.5654	0.0001	e	36	1	PS(P-18:0/18:0)	[M-H]-	49	40
800.5811	800.5811	0.0000	e	38	2	PS(e38:2)	[M-H]-	46	33
770.5334	770.5341	0.0007	e	36	3	PS(e36:3)	[M-H]-	16	18
788.5443	788.5447	0.0004	d	36	1	PS(36:1)	[M-H]-	13	12
802.5962	802.5967	0.0005	e	38	1	PS(e38:1)	[M-H]-	12	17

Biology Open • Supplementary information

786.5286	786.5290	0.0004	d	36	2	PS(36:2)	[M-H]-	10	11
834.5284	834.5290	0.0006	d	40	6	PS(40:6)	[M-H]-	6	7
812.5440	812.5447	0.0007	d	38	3	PS(38:3)	[M-H]-	6	5
842.5913	842.5916	0.0003	d	40	2	PS(40:2)	[M-H]-	5	9
816.5756	816.5760	0.0004	d	38	1	PS(38:1)	[M-H]-	4	12
830.6275	830.6280	0.0005	e	40	1	PS(e40:1)	[M-H]-	4	7
814.5602	814.5603	0.0001	d	38	2	PS(38:2)	[M-H]-	4	8
836.5446	836.5447	0.0001	d	40	5	PS(40:5)	[M-H]-	4	4
840.5757	840.5760	0.0003	d	40	3	PS(40:3)	[M-H]-	3	5
828.6114	828.6124	0.0010	e	40	2	PS(e40:2)	[M-H]-	2	3
758.5330	758.5341	0.0011	e	35	2	PS(e35:2)	[M-H]-	2	2
796.5473	796.5498	0.0025	e	38	4	PS(e38:4)	[M-H]-	2	12
810.5281	810.5290	0.0009	d	38	4	PS(38:4)	[M-H]-	2	2
826.5965	826.5967	0.0002	e	40	3	PS(e40:3)	[M-H]-	2	1
838.5600	838.5603	0.0003	d	40	4	PS(40:4)	[M-H]-	2	2
864.5758	864.5760	0.0002	d	42	5	PS(42:5)	[M-H]-	2	7
866.5915	866.5916	0.0001	d	42	4	PS(42:4)	[M-H]-	1	6
818.5911	818.5917	0.0006	d	38	0	PS(38:0)	[M-H]-	1	4
756.5181	756.5185	0.0004	e	35	3	PS(e35:3)	[M-H]-	1	2
868.6070	868.6073	0.0003	d	42	3	PS(42:3)	[M-H]-	1	4
890.5904	890.5916	0.0012	d	44	6	PS(44:6)	[M-H]-	1	3
860.5443	860.5447	0.0004	d	42	7	PS(42:7)	[M-H]-	1	3
824.5805	824.5811	0.0006	e	40	4	PS(e40:4)	[M-H]-	1	<1
870.6216	870.6229	0.0013	d	42	2	PS(42:2)	[M-H]-	1	2
892.6064	892.6073	0.0009	d	44	5	PS(44:5)	[M-H]-	1	2
784.5127	784.5134	0.0007	d	36	3	PS(36:3)	[M-H]-	<1	<1
872.6382	872.6386	0.0004	d	42	1	PS(42:1)	[M-H]-	<1	1
818.5337	818.5341	0.0004	e	40	7	PS(e40:7)	[M-H]-	<1	1
888.5753	888.5760	0.0007	d	44	7	PS(44:7)	[M-H]-	<1	1
804.6129	804.6123	0.0006	e	38	0	PS(e38:0)	[M-H]-	<1	1
790.5596	790.5603	0.0007	d	36	0	PS(36:0)	[M-H]-	<1	1
894.6224	894.6229	0.0005	d	44	4	PS(44:4)	[M-H]-	<1	1
742.5021	742.5028	0.0007	e	34	3	PS(34:3)	[M-H]-	<1	1
808.5113	808.5123	0.0010	d	38	5	PS(38:5)	[M-H]-	<1	<1
832.5134	832.5134	0.0000	d	40	7	PS(40:7)	[M-H]-	<1	<1
822.5629	822.5654	0.0025	e	40	5	PS(e40:5)	[M-H]-	<1	<1
766.5026	766.5028	0.0002	e	36	5	PS(e36:5)	[M-H]-	<1	2
744.5174	744.5185	0.0011	e	34	2	PS(e34:2)	[M-H]-	<1	<1
740.4878	740.4872	0.0006	e	34	4	PS(e34:4)	[M-H]-	<1	<1
790.5027	790.5028	0.0001	e	38	7	PS(e38:7)	[M-H]-	<1	<1

Biology Open • Supplementary information

Table S5: PG, negative ionisation mode

Input Mass	Matched Mass	Δ	type*	C	DB	Species	Ion	% Flagella	% Whole cells
773.5330	773.5338	0.0008	d	36	2	PG(18:0/18:2)	[M-H]-	100	100
759.5540	759.5545	0.0005	e	36	2	PG(O-18:0/18:2)	[M-H]-	77	59
775.5494	775.5494	0.0000	d	36	1	PG(18:0/18:1)	[M-H]-	43	35
757.5379	757.5378	0.0001	e	36	3	PG(e36:3)	[M-H]-	33	11
821.5329	821.5338	0.0009	d	40	6	PG(18:0/22:6)	[M-H]-	26	25
771.5179	771.5181	0.0002	d	36	3	PG(18:1/18:2)	[M-H]-	16	21
823.5488	823.5494	0.0006	d	40	5	PG(40:5)	[M-H]-	11	12
817.5020	817.5025	0.0005	d	40	8	PG(18:2/22:6)	[M-H]-	9	10
769.5022	769.5025	0.0003	d	36	4	PG(18:2/18:2)	[M-H]-	8	11
819.5179	819.5181	0.0002	d	40	7	PG(18:1/22:6);(18:2/22:5)	[M-H]-	9	11
865.5016	865.5025	0.0009	d	44	12	PG(44:12)	[M-H]-	4	6
747.5177	747.5181	0.0004	d	34	1	PG(34:1)	[M-H]-	3	3
867.5175	867.5182	0.0007	d	44	10	PG(44:10)	[M-H]-	2	4
745.5022	745.5025	0.0003	d	34	2	PG(34:2)	[M-H]-	4	6
841.5017	841.5025	0.0008	d	42	10	PG(42:10)	[M-H]-	1	1
843.5176	843.5181	0.0005	d	42	9	PG(42:9)	[M-H]-	1	1
743.4859	743.4868	0.0009	d	34	3	PG(34:3)	[M-H]-	<1	1
869.5330	869.5338	0.0008	d	44	10	PG(44:10)	[M-H]-	<1	1

Biology Open • Supplementary information

Table S6: SM, positive ionisation mode

Input Mass	Matched Mass	Δ	C	DB	Species	Ion	% Flagella	% Whole cells
675.5440	675.5435	0.0005	32	1	SM(d32:1)	[M+H]+	100	100
703.5750	703.5749	0.0001	34	1	SM(d34:1)	[M+H]+	96	99
717.5907	717.5905	0.0002	35	1	SM(d35:1)	[M+H]+	29	20
731.6064	731.6062	0.0002	36	1	SM(d36:1)	[M+H]+	34	19
705.5909	705.5905	0.0004	34	0	SM(d34:0)	[M+H]+	34	19
677.5597	677.5562	0.0035	32	0	SM(d32:0)	[M+H]+	25	15
689.5596	689.5592	0.0004	33	1	SM(d33:1)	[M+H]+	16	11
719.6068	719.6061	0.0007	35	0	SM(d35:0)	[M+H]+	13	6
733.6223	733.6218	0.0005	36	0	SM(d36:0)	[M+H]+	11	5
701.5596	701.5592	0.0004	34	2	SM(d34:2)	[M+H]+	5	5
729.5906	729.5905	0.0001	36	2	SM(d36:2)	[M+H]+	3	3

SM, negative ionisation mode

Input Mass	Matched Mass	Δ	C	DB	Species	Ion	% Flagella	% Whole cells
747.5655	747.5653	0.0002	34	1	SM(d34:1)	[M+Formate]-	100	100
719.5336	719.5340	0.0004	32	1	SM(d32:1)	[M+Formate]-	86	94
749.5813	749.5810	0.0003	34	0	SM(d34:0)	[M+Formate]-	25	22
761.5809	761.5810	0.0001	35	1	SM(d35:1)	[M+Formate]-	18	20
775.5966	775.5966	0.0000	36	1	SM(d36:1)	[M+Formate]-	14	14

Biology Open • Supplementary information

Table S7: IPC, positive ionisation mode

Input Mass	Matched Mass	Δ	C	DB	Species	Ion	% Flagella	% Whole cells
780.5392	780.5385	0.0007	34	1	IPC(34:1)	[M+H]+	100	100
752.5075	752.5072	0.0003	32	1	IPC(32:1)	[M+H]+	59	34
794.5547	794.5542	0.0005	35	1	IPC(35:1)	[M+H]+	20	23
782.5564	782.5542	0.0022	34	0	IPC(34:0)	[M+H]+	21	17
754.5243	754.5229	0.0014	32	0	IPC(32:0)	[M+H]+	17	10
766.5230	766.5229	0.0001	33	1	IPC(33:1)	[M+H]+	6	9

IPC, negative ionisation mode

Input Mass	Matched Mass	Δ	C	DB	Species	Ion	% Flagella	% Whole cells
778.5234	778.5240	0.0006	34	1	IPC(34:1)	[M-H]-	100	100
750.4922	750.4921	0.0001	32	1	IPC(32:1)	[M-H]-	68	50
752.5086	752.5099	0.0013	32	0	IPC(32:0)	[M-H]-	25	19
792.5388	792.5396	0.0008	35	0	IPC(35:1)	[M-H]-	19	24
796.5340	796.5345	0.0005	34	0	IPC(t34:0)	[M-H]-	17	2
780.5389	780.5396	0.0007	34	0	IPC(34:0)	[M-H]-	15	15
764.5077	764.5083	0.0006	35	1	IPC(35:1)	[M-H]-	14	11
806.5543	806.5547	0.0004	36	1	IPC(36:1)	[M-H]-	13	16
794.5545	794.5553	0.0008	35	1	IPC(35:0)	[M-H]-	6	6
776.5071	776.5083	0.0012	34	2	IPC(34:2)	[M-H]-	5	5
808.5725	808.5705	0.0020	36	0	IPC(36:0)	[M-H]-	4	3
766.5236	766.5240	0.0004	33	0	IPC(33:0)	[M-H]-	4	3

Biology Open • Supplementary information

Table S8: Ceramide, positive ionisation mode

Input Mass	Matched Mass	Δ	C	DB	Species	Ion	% Flagella	% Whole cells
510.4885	510.4881	0.0004	32	1	Cer(d32:1)	[M+H]+	100	100
538.5196	538.5194	0.0002	34	1	Cer(d34:1)	[M+H]+	96	90
566.5510	566.5507	0.0003	18	1	Cer(d36:1)	[M+H]+	79	35
568.5666	568.5663	0.0003	36	0	Cer(d36:0)	[M+H]+	51	18
540.5352	540.5350	0.0002	34	0	Cer(d34:0)	[M+H]+	45	18
512.5040	512.5037	0.0003	32	0	Cer(d32:0)	[M+H]+	45	33
300.2904	300.2897	0.0007	18	1	Cer(d18:1)	[M+H]+	43	21

Biology Open • Supplementary information
